# [μ-*N*,*N*,*N*′,*N*′-Tetra­kis(2-pyridyl­meth­yl)butane-1,4-diamine]­bis­[dichlorido­copper(II)] trihydrate

**DOI:** 10.1107/S1600536810034501

**Published:** 2010-09-04

**Authors:** Mark Bartholomä, Hoi Cheung, Jon Zubieta

**Affiliations:** a Department of Chemistry, Syracuse University, Syracuse, New York 13244, USA

## Abstract

The title dinuclear copper complex, [Cu_2_Cl_4_(C_28_H_32_N_6_)]·3H_2_O, is located on a crystallographic inversion center. The unique Cu^II^ ion is coordinated in a slightly distorted square-pyramidal environment in which the N atoms of the dipicolyl­amine group and a chloride ligand form the basal plane. The apical position is occupied by a second chloride atom. While the Cu—N distances of the pyridine N atoms are the same within expermental error, the Cu—N distance to the tertiary N atom is slightly elongated. The apical Cu—Cl distance is elongated due to typical Jahn–Teller distortion. One of the water O atoms was refined as disordered over two sites with occupancies 0.734 (17):0.266 (17) and another with half occupancy. H atoms for the disordered solvent atoms were not included in the refinement.

## Related literature

For crystallographic data of tetra­kis­(pyridin-2-yl-meth­yl)alkyl-diamines, see: Fujihara *et al.* (2004 ▶); Mambanda *et al.* (2007 ▶). For the superoxide dismutase activity of iron complexes, see: Tamura *et al.* (2000 ▶). For dinuclear Pt complexes of similar ligands, see: Ertürk *et al.* (2007 ▶). For the use of the dipicolyl­amine moiety for binding of the *M*(CO)_3_ core (*M* = Re, ^99*m*^Tc), see: Bartholomä *et al.* (2009 ▶). For crystal structures closely related to the title compound, see: Bartholomä *et al.* (2010*a* ▶,*b* ▶,*c* ▶,*d* ▶).
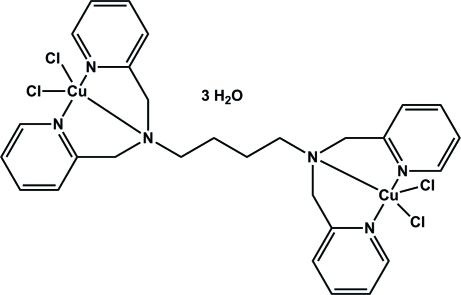


## Experimental

### 

#### Crystal data

[Cu_2_Cl_4_(C_28_H_32_N_6_)]·3H_2_O
*M* *_r_* = 775.52
Monoclinic, 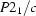

*a* = 11.4403 (5) Å
*b* = 10.0230 (5) Å
*c* = 14.2943 (7) Å
β = 106.143 (1)°
*V* = 1574.44 (13) Å^3^
*Z* = 2
Mo *K*α radiation
μ = 1.73 mm^−1^
*T* = 90 K
0.26 × 0.18 × 0.14 mm

#### Data collection

Bruker APEX CCD diffractometer
Absorption correction: multi-scan (*SADABS*; Sheldrick, 1996 ▶) *T*_min_ = 0.662, *T*_max_ = 0.794
15464 measured reflections
3906 independent reflections
3746 reflections with *I* > 2σ(*I*)
*R*_int_ = 0.027

#### Refinement

*R*[*F* ^2^ > 2σ(*F* ^2^)] = 0.053
*wR*(*F* ^2^) = 0.125
*S* = 1.25
3906 reflections
209 parameters
H-atom parameters constrained
Δρ_max_ = 0.76 e Å^−3^
Δρ_min_ = −0.49 e Å^−3^

### 

Data collection: *SMART* (Bruker, 2002 ▶); cell refinement: *SAINT* (Bruker, 2002 ▶); data reduction: *SAINT*; program(s) used to solve structure: *SHELXS97* (Sheldrick, 2008 ▶); program(s) used to refine structure: *SHELXL97* (Sheldrick, 2008 ▶); molecular graphics: *DIAMOND* (Brandenburg & Putz, 1999 ▶); software used to prepare material for publication: *SHELXTL* (Sheldrick, 2008 ▶).

## Supplementary Material

Crystal structure: contains datablocks I, global. DOI: 10.1107/S1600536810034501/lh5107sup1.cif

Structure factors: contains datablocks I. DOI: 10.1107/S1600536810034501/lh5107Isup2.hkl

Additional supplementary materials:  crystallographic information; 3D view; checkCIF report

## Figures and Tables

**Table 1:** Selected bond lengths (Å)

Cu1—N2	2.011 (3)
Cu1—N3	2.016 (3)
Cu1—N1	2.064 (3)
Cu1—Cl2	2.2532 (8)
Cu1—Cl1	2.5612 (10)

**Fig. 1.**
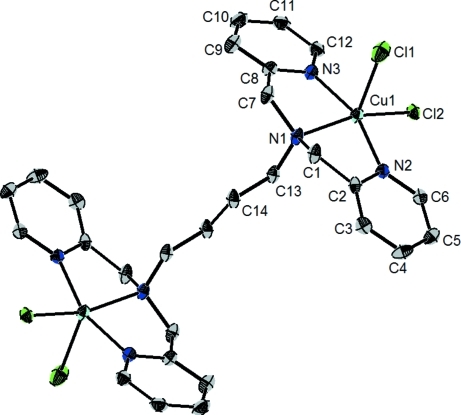
The molecular structure of the title complex. The displacement ellipsoids are drawn at 50% probability level. Solvent water and hydrogen atoms are omitted for clarity. Unlabeled atoms are related by the symmetry code (-x + 1, -y + 1, -z + 1).

## supplementary crystallographic information

### Comment

The described ligand has been used as starting material for hydrothermal synthesis of metal-organic transition metal/molybdateoxide frameworks in the principal author's laboratory (Bartholomä, unpublished results). The dipicolylamine moiety has originally been developed in our laboratory as metal chelating entity for binding of the *M*(CO)_3_ core (*M* = Re,^99^*^m^*Tc) for radiopharmaceutical purposes. However, a different coordination mode has been observed for the *M*(CO)_3_ core in which the dipicolylamine metal chelate is coordinated in a facial manner (Bartholomä, 2009).

The title complex was prepared as part of a series with different cadmium and copper salts to study the coordination properties of the ligand with these metals without the interaction of metaloxide clusters (Bartholomä, 2010*a*,b). The use of copper bromide as metal salt gave a structurally comparable complex with a square pyramidal coordination sphere of both copper atoms (Bartholomä, 2010*c*). The Cu—N_py_ distances were determined to 2.015 (6) Å and 2.019 (5) Å, and the Cu—N*_tert_* distance is 2.053 (5) Å. The extension of the spacer between the two dipicolylamine moieties in the case of *N^1^*,*N^1^*,*N^5^*,*N^5^*-tetrakis(pyridin-2-ylmethyl)pentane-1,5-diamine with copper chloride also resulted in a structurally similar complex with Cu—N_py_ distances of 1.986 (4) Å and 1.996 (4) Å, and a Cu—N*_tert_* distance of 2.077 (4) Å (Bartholomä *et al.*, 2010d).

Crystal structures of the ligands *N^1^*,*N^1^*,*N^3^*,*N^3^*-tetrakis(2-pyridiniomethyl)-1,3-diaminopropane and *N^1^*,*N^1^*,*N^4^*,*N^4^*-tetrakis(pyridin-2-ylmethyl)butane-1,4-diamine have been described recently (Fujihara, 2004; Mambanda, 2007). Superoxide dismutase activity of iron(II) complexes of *N^1^*,*N^1^*,*N^3^*,*N^3^*-tetrakis(2-pyridiniomethyl)-1,3-diaminopropane and related ligands has been investigated by Tamura *et al.* (2000). Studies on the thermodynamic and kinetic behaviour of the reaction of platinum(II) complexes of higher ligand homologues with chloride have been performed by Ertürk *et al.* (2007).

### Experimental

*N^1^*,*N^1^*,*N^4^*,*N^4^*-tetrakis(pyridin-2-ylmethyl)butane-1,4-diamine. An amount of 1.00 g (11.34 mmol) 1,4-diaminobutane was dissolved in 30 ml anhydrous dichloroethane under an inert atmosphere (argon) followed by the addition of 4.55 ml (47.65 mmol) pyridine-2-carboxaldehyde. The mixture was stirred for 15 min at r.t. and then cooled with an ice bath prior to the portionwise addition of 14.43 g (68.06 mmol) sodium triacetoxyborohydride (gas evolution, exothermic reaction). The reaction was stirred overnight allowing the temperature slowly to rise to room temperature. The reaction was quenched by the dropwise addition of saturated sodium bicarbonate solution and stirring was continued until the gas evolution ceased. The mixture was separated and the organic layer was further washed with saturated sodium bicarbonate solution, water and brine. The organic phase was dried with anhydrous sodium sulfate, filtered and the solvent removed under reduced pressure. The crude reaction mixture was then purified by silica gel column chromatography starting with chloroform and increasing gradient to chloroform:methanol 10:1 (*v*/*v*). Yield: 4.02 g (78%). ^1^H NMR (CDCl_3_): *δ* = 8.40 (m, 4H), 7.51 (m, 4H), 7.39 (d, J = 7.81 Hz, 4H), 7.02 (m, 4H), 3.67 (s, 8H), 2.39 (m, 4H), 1.42 (m, 4H) p.p.m..

Synthesis of metal complex. To 2 ml of an aqueous solution of copper chloride, two equivalents (50 mg, 0.11 mmol) of *N^1^*,*N^1^*,*N^4^*,*N^4^*-tetrakis(pyridin-2-ylmethyl)butane-1,4-diamine in 2 ml methanol were added followed by the addition of 2 ml *N*,*N*-dimethylformamide. Single crystals were obtained after a week by slow evaporation of the solvents at room temperature.

### Refinement

All the *C*—H atoms were placed in idealized positions and refined in a riding-model approximation with *C*—H_aryl_ = 0.95, *C*—H_methyl_ = 0.98 and *C*—H_methylene_ = 0.99Å and *U*_iso_(H) = 1.5*U*_eq_(C_methyl_) and 1.2*U*_eq_(C_methylene/aryl_). The water H atoms were not included in the refinement.

### Crystal data

**Table:**

[Cu_2_Cl_4_(C_28_H_32_N_6_)]·3H_2_O	*F*(000) = 796
*M**_r_* = 775.52	*D*_x_ = 1.636 Mg m^−^^3^
Monoclinic, *P*2_1_/*c*	Mo *K*α radiation, λ = 0.71073 Å
Hall symbol: -P 2ybc	Cell parameters from 5514 reflections
*a* = 11.4403 (5) Å	θ = 2.5–28.2°
*b* = 10.0230 (5) Å	µ = 1.73 mm^−^^1^
*c* = 14.2943 (7) Å	*T* = 90 K
β = 106.143 (1)°	Block, blue
*V* = 1574.44 (13) Å^3^	0.26 × 0.18 × 0.14 mm
*Z* = 2	

### Data collection

**Table:**

Bruker APEX CCD diffractometer	3906 independent reflections
Radiation source: fine-focus sealed tube	3746 reflections with *I* > 2σ(*I*)
graphite	*R*_int_ = 0.027
Detector resolution: 512 pixels mm^-1^	θ_max_ = 28.3°, θ_min_ = 1.9°
φ and ω scans	*h* = −15→13
Absorption correction: multi-scan (*SADABS*; Sheldrick, 1996)	*k* = −13→12
*T*_min_ = 0.662, *T*_max_ = 0.794	*l* = −19→19
15464 measured reflections	

### Refinement

**Table:**

Refinement on *F*^2^	Primary atom site location: structure-invariant direct methods
Least-squares matrix: full	Secondary atom site location: difference Fourier map
*R*[*F*^2^ > 2σ(*F*^2^)] = 0.053	Hydrogen site location: inferred from neighbouring sites
*wR*(*F*^2^) = 0.125	H-atom parameters constrained
*S* = 1.25	*w* = 1/[σ^2^(*F*_o_^2^) + (0.0448*P*)^2^ + 3.8247*P*] where *P* = (*F*_o_^2^ + 2*F*_c_^2^)/3
3906 reflections	(Δ/σ)_max_ < 0.001
209 parameters	Δρ_max_ = 0.76 e Å^−^^3^
0 restraints	Δρ_min_ = −0.49 e Å^−^^3^

### Special details

**Table:**

Geometry. All e.s.d.'s (except the e.s.d. in the dihedral angle between two l.s. planes) are estimated using the full covariance matrix. The cell e.s.d.'s are taken into account individually in the estimation of e.s.d.'s in distances, angles and torsion angles; correlations between e.s.d.'s in cell parameters are only used when they are defined by crystal symmetry. An approximate (isotropic) treatment of cell e.s.d.'s is used for estimating e.s.d.'s involving l.s. planes.
Refinement. Refinement of *F*^2^ against ALL reflections. The weighted *R*-factor *wR* and goodness of fit *S* are based on *F*^2^, conventional *R*-factors *R* are based on *F*, with *F* set to zero for negative *F*^2^. The threshold expression of *F*^2^ > σ(*F*^2^) is used only for calculating *R*-factors(gt) *etc*. and is not relevant to the choice of reflections for refinement. *R*-factors based on *F*^2^ are statistically about twice as large as those based on *F*, and *R*- factors based on ALL data will be even larger.

### Fractional atomic coordinates and isotropic or equivalent isotropic displacement parameters (Å^2^)

**Table:**

	*x*	*y*	*z*	*U*_iso_*/*U*_eq_	Occ. (<1)
Cu1	0.66753 (3)	0.34837 (4)	0.83401 (3)	0.01782 (12)	
Cl1	0.75297 (10)	0.11459 (9)	0.88332 (8)	0.0361 (2)	
Cl2	0.68782 (7)	0.45176 (8)	0.97763 (5)	0.01982 (17)	
O1A	0.0560 (9)	0.1843 (9)	0.0017 (4)	0.077 (3)	0.734 (17)
O1B	−0.0110 (12)	0.2423 (13)	0.0088 (8)	0.034 (4)	0.266 (17)
O2	0.1966 (10)	0.1197 (11)	0.9798 (7)	0.091 (3)	0.50
N1	0.6228 (2)	0.3205 (3)	0.68520 (19)	0.0180 (5)	
N2	0.8238 (2)	0.4118 (3)	0.81070 (19)	0.0191 (5)	
N3	0.4893 (2)	0.3059 (3)	0.80922 (19)	0.0173 (5)	
C1	0.7378 (3)	0.2849 (3)	0.6642 (2)	0.0226 (7)	
H1A	0.7299	0.2968	0.5940	0.027*	
H1B	0.7581	0.1903	0.6813	0.027*	
C2	0.8365 (3)	0.3741 (3)	0.7235 (2)	0.0205 (6)	
C3	0.9347 (3)	0.4152 (4)	0.6916 (3)	0.0281 (7)	
H3	0.9411	0.3895	0.6292	0.034*	
C4	1.0234 (3)	0.4943 (4)	0.7523 (3)	0.0296 (8)	
H4	1.0916	0.5235	0.7321	0.036*	
C5	1.0118 (3)	0.5302 (4)	0.8424 (3)	0.0259 (7)	
H5	1.0723	0.5833	0.8853	0.031*	
C6	0.9106 (3)	0.4878 (4)	0.8695 (2)	0.0227 (7)	
H6	0.9023	0.5131	0.9314	0.027*	
C7	0.5324 (3)	0.2111 (3)	0.6684 (2)	0.0216 (7)	
H7A	0.5728	0.1257	0.6930	0.026*	
H7B	0.4918	0.2014	0.5979	0.026*	
C8	0.4408 (3)	0.2469 (3)	0.7223 (2)	0.0187 (6)	
C9	0.3169 (3)	0.2237 (3)	0.6863 (2)	0.0220 (6)	
H9	0.2841	0.1849	0.6239	0.026*	
C10	0.2420 (3)	0.2587 (4)	0.7439 (3)	0.0269 (7)	
H10	0.1569	0.2427	0.7216	0.032*	
C11	0.2915 (3)	0.3166 (3)	0.8337 (3)	0.0237 (7)	
H11	0.2415	0.3400	0.8742	0.028*	
C12	0.4152 (3)	0.3399 (3)	0.8636 (2)	0.0192 (6)	
H12	0.4492	0.3815	0.9248	0.023*	
C13	0.5697 (3)	0.4462 (3)	0.6335 (2)	0.0188 (6)	
H13A	0.6345	0.5146	0.6450	0.023*	
H13B	0.5062	0.4793	0.6626	0.023*	
C14	0.5139 (3)	0.4315 (3)	0.5237 (2)	0.0208 (7)	
H14A	0.5712	0.3832	0.4951	0.025*	
H14B	0.4379	0.3787	0.5110	0.025*	

### Atomic displacement parameters (Å^2^)

**Table:**

	*U*^11^	*U*^22^	*U*^33^	*U*^12^	*U*^13^	*U*^23^
Cu1	0.0181 (2)	0.0213 (2)	0.0155 (2)	−0.00268 (15)	0.00696 (14)	−0.00284 (14)
Cl1	0.0447 (6)	0.0233 (4)	0.0501 (6)	0.0064 (4)	0.0293 (5)	0.0045 (4)
Cl2	0.0218 (4)	0.0235 (4)	0.0137 (3)	−0.0005 (3)	0.0042 (3)	−0.0016 (3)
O1A	0.085 (6)	0.085 (6)	0.059 (4)	−0.025 (5)	0.017 (3)	−0.014 (3)
O1B	0.035 (7)	0.039 (7)	0.026 (5)	0.015 (5)	0.003 (4)	0.010 (4)
O2	0.100 (8)	0.088 (7)	0.079 (7)	0.005 (6)	0.016 (6)	0.005 (5)
N1	0.0201 (13)	0.0188 (13)	0.0173 (12)	−0.0048 (10)	0.0086 (10)	−0.0066 (10)
N2	0.0183 (13)	0.0212 (13)	0.0196 (12)	0.0002 (10)	0.0081 (10)	0.0003 (11)
N3	0.0184 (12)	0.0184 (13)	0.0155 (12)	−0.0006 (10)	0.0051 (10)	0.0001 (10)
C1	0.0272 (17)	0.0220 (16)	0.0229 (15)	−0.0014 (13)	0.0141 (13)	−0.0046 (13)
C2	0.0197 (15)	0.0228 (16)	0.0207 (15)	0.0024 (12)	0.0084 (12)	0.0004 (12)
C3	0.0267 (18)	0.0318 (19)	0.0307 (18)	0.0006 (15)	0.0162 (14)	−0.0020 (15)
C4	0.0191 (17)	0.033 (2)	0.040 (2)	0.0004 (14)	0.0135 (15)	0.0052 (16)
C5	0.0160 (15)	0.0293 (18)	0.0290 (17)	−0.0005 (13)	0.0005 (13)	0.0060 (14)
C6	0.0205 (16)	0.0254 (17)	0.0204 (15)	−0.0004 (13)	0.0026 (12)	0.0019 (13)
C7	0.0270 (17)	0.0206 (16)	0.0197 (15)	−0.0077 (13)	0.0108 (12)	−0.0062 (12)
C8	0.0236 (16)	0.0159 (14)	0.0179 (14)	−0.0023 (12)	0.0076 (12)	0.0007 (11)
C9	0.0236 (16)	0.0202 (16)	0.0202 (14)	−0.0041 (12)	0.0031 (12)	−0.0040 (12)
C10	0.0175 (15)	0.0249 (17)	0.0376 (19)	−0.0023 (13)	0.0063 (14)	−0.0069 (15)
C11	0.0232 (16)	0.0215 (16)	0.0287 (17)	0.0008 (13)	0.0109 (13)	−0.0030 (13)
C12	0.0228 (16)	0.0177 (15)	0.0175 (14)	0.0000 (12)	0.0063 (12)	0.0004 (11)
C13	0.0228 (15)	0.0186 (15)	0.0171 (14)	−0.0040 (12)	0.0095 (12)	−0.0033 (11)
C14	0.0253 (16)	0.0242 (17)	0.0130 (13)	−0.0068 (13)	0.0056 (12)	−0.0053 (12)

### Geometric parameters (Å, °)

**Table:**

Cu1—N2	2.011 (3)	C5—C6	1.386 (5)
Cu1—N3	2.016 (3)	C5—H5	0.9500
Cu1—N1	2.064 (3)	C6—H6	0.9500
Cu1—Cl2	2.2532 (8)	C7—C8	1.506 (4)
Cu1—Cl1	2.5612 (10)	C7—H7A	0.9900
N1—C1	1.473 (4)	C7—H7B	0.9900
N1—C7	1.480 (4)	C8—C9	1.387 (5)
N1—C13	1.501 (4)	C9—C10	1.389 (5)
N2—C6	1.345 (4)	C9—H9	0.9500
N2—C2	1.348 (4)	C10—C11	1.379 (5)
N3—C12	1.344 (4)	C10—H10	0.9500
N3—C8	1.348 (4)	C11—C12	1.379 (5)
C1—C2	1.504 (5)	C11—H11	0.9500
C1—H1A	0.9900	C12—H12	0.9500
C1—H1B	0.9900	C13—C14	1.527 (4)
C2—C3	1.388 (5)	C13—H13A	0.9900
C3—C4	1.387 (5)	C13—H13B	0.9900
C3—H3	0.9500	C14—C14^i^	1.526 (7)
C4—C5	1.378 (5)	C14—H14A	0.9900
C4—H4	0.9500	C14—H14B	0.9900
			
N2—Cu1—N3	160.10 (11)	C4—C5—H5	120.5
N2—Cu1—N1	81.33 (11)	C6—C5—H5	120.5
N3—Cu1—N1	80.87 (11)	N2—C6—C5	121.9 (3)
N2—Cu1—Cl2	97.73 (8)	N2—C6—H6	119.1
N3—Cu1—Cl2	95.75 (8)	C5—C6—H6	119.1
N1—Cu1—Cl2	159.16 (8)	N1—C7—C8	107.1 (3)
N2—Cu1—Cl1	92.63 (8)	N1—C7—H7A	110.3
N3—Cu1—Cl1	98.35 (8)	C8—C7—H7A	110.3
N1—Cu1—Cl1	97.23 (8)	N1—C7—H7B	110.3
Cl2—Cu1—Cl1	103.61 (3)	C8—C7—H7B	110.3
C1—N1—C7	114.3 (3)	H7A—C7—H7B	108.5
C1—N1—C13	111.4 (2)	N3—C8—C9	122.1 (3)
C7—N1—C13	111.9 (3)	N3—C8—C7	114.2 (3)
C1—N1—Cu1	105.3 (2)	C9—C8—C7	123.7 (3)
C7—N1—Cu1	103.27 (19)	C8—C9—C10	118.3 (3)
C13—N1—Cu1	110.15 (18)	C8—C9—H9	120.9
C6—N2—C2	119.1 (3)	C10—C9—H9	120.9
C6—N2—Cu1	127.7 (2)	C11—C10—C9	119.8 (3)
C2—N2—Cu1	113.2 (2)	C11—C10—H10	120.1
C12—N3—C8	118.7 (3)	C9—C10—H10	120.1
C12—N3—Cu1	128.0 (2)	C12—C11—C10	118.7 (3)
C8—N3—Cu1	113.0 (2)	C12—C11—H11	120.7
N1—C1—C2	108.3 (3)	C10—C11—H11	120.7
N1—C1—H1A	110.0	N3—C12—C11	122.4 (3)
C2—C1—H1A	110.0	N3—C12—H12	118.8
N1—C1—H1B	110.0	C11—C12—H12	118.8
C2—C1—H1B	110.0	N1—C13—C14	114.9 (3)
H1A—C1—H1B	108.4	N1—C13—H13A	108.5
N2—C2—C3	121.7 (3)	C14—C13—H13A	108.5
N2—C2—C1	115.5 (3)	N1—C13—H13B	108.5
C3—C2—C1	122.7 (3)	C14—C13—H13B	108.5
C4—C3—C2	118.8 (3)	H13A—C13—H13B	107.5
C4—C3—H3	120.6	C14^i^—C14—C13	110.2 (3)
C2—C3—H3	120.6	C14^i^—C14—H14A	109.6
C5—C4—C3	119.4 (3)	C13—C14—H14A	109.6
C5—C4—H4	120.3	C14^i^—C14—H14B	109.6
C3—C4—H4	120.3	C13—C14—H14B	109.6
C4—C5—C6	119.1 (3)	H14A—C14—H14B	108.1
			
N2—Cu1—N1—C1	32.6 (2)	Cu1—N2—C2—C3	175.9 (3)
N3—Cu1—N1—C1	−156.3 (2)	C6—N2—C2—C1	177.7 (3)
Cl2—Cu1—N1—C1	121.5 (2)	Cu1—N2—C2—C1	−4.3 (4)
Cl1—Cu1—N1—C1	−59.0 (2)	N1—C1—C2—N2	32.3 (4)
N2—Cu1—N1—C7	152.7 (2)	N1—C1—C2—C3	−147.9 (3)
N3—Cu1—N1—C7	−36.2 (2)	N2—C2—C3—C4	1.6 (5)
Cl2—Cu1—N1—C7	−118.3 (2)	C1—C2—C3—C4	−178.1 (3)
Cl1—Cu1—N1—C7	61.1 (2)	C2—C3—C4—C5	−0.1 (6)
N2—Cu1—N1—C13	−87.6 (2)	C3—C4—C5—C6	−0.9 (5)
N3—Cu1—N1—C13	83.5 (2)	C2—N2—C6—C5	1.0 (5)
Cl2—Cu1—N1—C13	1.3 (4)	Cu1—N2—C6—C5	−176.7 (3)
Cl1—Cu1—N1—C13	−179.20 (18)	C4—C5—C6—N2	0.5 (5)
N3—Cu1—N2—C6	134.7 (3)	C1—N1—C7—C8	162.1 (3)
N1—Cu1—N2—C6	161.4 (3)	C13—N1—C7—C8	−70.1 (3)
Cl2—Cu1—N2—C6	2.5 (3)	Cu1—N1—C7—C8	48.3 (3)
Cl1—Cu1—N2—C6	−101.6 (3)	C12—N3—C8—C9	1.8 (5)
N3—Cu1—N2—C2	−43.1 (5)	Cu1—N3—C8—C9	−172.4 (3)
N1—Cu1—N2—C2	−16.4 (2)	C12—N3—C8—C7	−179.0 (3)
Cl2—Cu1—N2—C2	−175.3 (2)	Cu1—N3—C8—C7	6.9 (3)
Cl1—Cu1—N2—C2	80.5 (2)	N1—C7—C8—N3	−38.3 (4)
N2—Cu1—N3—C12	−129.5 (3)	N1—C7—C8—C9	140.9 (3)
N1—Cu1—N3—C12	−156.2 (3)	N3—C8—C9—C10	−2.2 (5)
Cl2—Cu1—N3—C12	3.0 (3)	C7—C8—C9—C10	178.6 (3)
Cl1—Cu1—N3—C12	107.7 (3)	C8—C9—C10—C11	0.9 (5)
N2—Cu1—N3—C8	44.0 (4)	C9—C10—C11—C12	0.8 (5)
N1—Cu1—N3—C8	17.2 (2)	C8—N3—C12—C11	0.0 (5)
Cl2—Cu1—N3—C8	176.5 (2)	Cu1—N3—C12—C11	173.2 (2)
Cl1—Cu1—N3—C8	−78.8 (2)	C10—C11—C12—N3	−1.3 (5)
C7—N1—C1—C2	−154.9 (3)	C1—N1—C13—C14	73.2 (3)
C13—N1—C1—C2	77.0 (3)	C7—N1—C13—C14	−56.1 (3)
Cu1—N1—C1—C2	−42.4 (3)	Cu1—N1—C13—C14	−170.4 (2)
C6—N2—C2—C3	−2.1 (5)	N1—C13—C14—C14^i^	−168.8 (3)

Symmetry codes: (i) −*x*+1, −*y*+1, −*z*+1.
